# Adherence and compliance rates across post-dieting strategies: a preliminary analysis

**DOI:** 10.1080/15502783.2025.2550186

**Published:** 2025-08-26

**Authors:** Valentina Rodriguez Da Silva, John Holtje, Ashlyn Bratton, Jakob Paz, Zia Rahaman, Eric T. Trexler, Vladyslava Martynovska, Sara Hobbes, Cassandra Resler, Sydney Monahan, Valeria V. Silva Ramirez, Bill I. Campbell

**Affiliations:** a University of South Florida, Performance & Physique Enhancement Lab, Exercise Science & Kinesiology Program, Tampa, FL, USA; b Duke University, Department of Evolutionary Anthropology, Durham, NC, USA

**Keywords:** Reverse diet, caloric intake, strategy, weight regain

## Abstract

**Background:**

There are a variety of post-diet strategies implemented to limit fat regain and maintain fat-free mass. This study investigated the adherence and attrition rates across three different post-diet strategies: a reverse diet (REV), an immediate return to maintenance (NIH), or an ad-libitum approach (CON).

**Methods:**

Resistance-trained subjects [75 total, 10 (13.3%) males, 65 (86.7%) females; 36.5 ± 6 years; 71.1 ± 13.9 kgs] underwent a 30% caloric deficit from a pre-determined maintenance. After achieving 5% bodyweight loss, subjects initiated their randomized post-diet strategy, which they followed for 15 weeks. The REV group underwent a gradual weekly caloric increase from the deficit (8.5% for males and 11.7% for females). The NIH group returned immediately to a newly calculated maintenance utilizing the NIH Bodyweight Planner. The CON group followed an ad-libitum diet. A chi-square test was used to compare the proportion of withdrawals among groups.

**Results:**

Seventy-five participants entered the post-diet phase and either withdrew or completed their assigned phase. Average prescribed calories differed significantly between REV and NIH (REV = 2423, NIH = 2179, *p* = 0.03367). Both groups under-consumed their prescribed calories by 259 and 177 calories, respectively, with no significant difference between groups (*p* = 0.2686). REV had a withdrawal rate of 45.2%, NIH of 30.4%, and CON of 23.8% with no significant differences between groups (*p* = 0.2488).

**Conclusion:**

It is believed that having a prescribed post-diet strategy may improve adherence and/or compliance in the post-diet period, but the present data challenges this assumption. Although caloric intake differed between REV and NIH by the end of the study, both groups under-consumed their prescribed intake, suggesting similar difficulties with adherence. Additionally, subject withdrawals were categorized into five categories: health-related causes, inability to follow study requirements, loss of contact with subject, personal reasons, and self-withdrawal. Given that there were no significant differences in withdrawal rates between the three groups, specific post-dieting strategies may not be directly predictive of compliance and, rather, other external variables may play a bigger role in determining post-dieting success. Further research may be needed to determine what these variables are, and whether there are additional benefits or detriments to these post-dieting strategies beyond adherence and compliance.

